# The mechanistic causes of peripheral intravenous catheter failure based on a parametric computational study

**DOI:** 10.1038/s41598-018-21617-1

**Published:** 2018-02-21

**Authors:** Russell Piper, Peter J. Carr, Lachlan J. Kelsey, Andrew C. Bulmer, Samantha Keogh, Barry J. Doyle

**Affiliations:** 1 Vascular Engineering Laboratory, Harry Perkins Institute of Medical Research, QEII Medical Centre, Nedlands and Centre for Medical Research, The University of Western Australia, Perth, Australia; 2 School of Engineering, The University of Western Australia, Perth, Australia; 3 Emergency Medicine, Faculty of Health and Medical Sciences, The University of Western Australia, Perth, Australia; 4 The Alliance for Vascular Access Teaching and Research Group, Menzies Health Institute Queensland, Griffith University, Brisbane, Australia; 5 School of Medical Science and Menzies Health Institute Queensland, Griffith University, Brisbane, Australia; 6 School of Nursing, Institute of Health and Biomedical Innovation, Queensland University of Technology, Brisbane, Australia; 7 BHF Centre for Cardiovascular Science, The University of Edinburgh, Edinburgh, UK

## Abstract

Peripheral intravenous catheters (PIVCs) are the most commonly used invasive medical device, yet up to 50% fail. Many pathways to failure are mechanistic and related to fluid mechanics, thus can be investigated using computational fluid dynamics (CFD). Here we used CFD to investigate typical PIVC parameters (infusion rate, catheter size, insertion angle and tip position) and report the hemodynamic environment (wall shear stress (WSS), blood damage, particle residence time and venous stasis volumes) within the vein and catheter, and show the effect of each PIVC parameter on each hemodynamic measure. Catheter infusion rate has the greatest impact on our measures, with catheter orientation also playing a significant role. In some PIVC configurations WSS was 3254 times higher than the patent vein, and blood damage was 512 times greater, when compared to control conditions. Residence time is geometry-dependent and decreases exponentially with increasing insertion angle. Stasis volume decreased with increasing infusion rate and, to a lesser degree, insertion angle. Even without infusion, the presence of the catheter changes the flow field, causing low velocity recirculation at the catheter tip. This research demonstrates how several controllable factors impact important mechanisms of PIVC failure. These data, the first of their kind, suggest limiting excessive infusion rates in PIVC.

## Introduction

The insertion of a peripheral intravenous catheter/cannula (PIVC) is the most common invasive medical procedure worldwide, with current annual estimates of over one billion devices used^1^. However, up to 50% of successfully inserted devices require removal due to failure prior to their clinical need being fulfilled^2^.

Clinical investigations describing failure mechanisms of PIVCs have been published^3^, resulting in interventional studies to update techniques for the securement of PIVCs^4^ and, in time, clinical guidelines^5^. Current PIVCs have two predominant failure ‘categories’; failure of insertion and failure after time *in situ*. Insertion failures, are largely influenced by the inserting clinician (assuming manufacturing standards are met)^1^.

*In situ* failure is associated with a triad of definitions some which are not mutually exclusive; (i) infiltration, i.e. where the infusion inadvertently escapes the vein lumen and/or is infused into the subcutaneous tissues^6^; (ii) occlusion, also referred to as blocked, where flushing or aspirating from the PIVC is not possible^7^; and (iii) phlebitis and/or thrombophlebitis^8^ leading to infection (either local or systemic), with systemic infection being particularly serious. Importantly, phlebitis is not always associated with thrombus formation, and can occur within the catheter potentially occluding due to fibrin deposition around the access port without any thrombus evident (catheter occlusion without vein occlusion), however, the two mechanisms are strongly inter-related. If phlebitis occurs first, particularly from vessel wall damage, activation of coagulation and inflammatory mediator release by the endothelium can trigger platelet aggregation and thrombus formation. If a thrombus forms first, particularly from stasis in flow, the processes that occur therein often inflame nearby biological tissues (such as the vessel wall) and cause phlebitis, similar to the mechanism that occurs in deep vein thrombosis.

Thrombosis in veins is commonly thought of in terms of Virchow’s Triad^9^ – a trio of broad categories of contributing factors to thrombosis formation *in situ*. (i) Hypercoagulability, usually related to patient-specific factors. (ii) Endothelial injury, which is an inevitable result of PIVC insertion and possibly caused by local physical and chemical stressors applied to the endothelium during catheter maintenance^10^. Additionally, recent findings show that if the tip of the catheter was near the wall of the vessel, this significantly increased the risk of subcutaneous oedema, likely associated with damage to, and phlebitis of the vessel wall^6^. (iii) Hemodynamic changes or venous stasis and turbulence.

Previous research investigated central venous catheters (CVCs) and reported an inconclusive link between turbulence and thrombus formation^11^. Nifong and McDevitt^12^ simulated a relationship in peripheral intravenous central catheters (PICCs) using a mathematical approach. They established that flow rates in a vein with a sited PICC can decrease by as much as 93%, and that this is proportional to the percentage of the vein lumen occupied by the device^12^. This theory also agrees with recent data reporting vein diameter should be greater than 3 mm to reduce risk of complication in PIVCs^13^. However, in contrast, a prospective cohort study by Sharp *et al*.^14^ produced a PICC to vein ratio and suggest that a target vein of 3 mm is an acceptable size for vein to accommodate a catheter diameter of 1.3 mm.

Several factors are involved in PIVC failure, some of which are patient and device specific. However, many of these important factors are directly influenced by the geometric configuration of the catheter and vein, in addition to flow conditions. A significant knowledge gap exists in the current literature concerning the impact of device geometry, angle of insertion, proximity to the endothelium and flushing speed on local hemodynamics. This study builds upon previous research^12^ by computationally analysing clinically relevant parameters in PIVCs. Although previous PICC data is applicable to PIVCs, to date, no study has comprehensively investigated the hemodynamics and shear stresses in veins with inserted PIVCs.

Computational fluid dynamics (CFD) has shown great potential in vascular research as it can be used to calculate approximately the WSS in any vascular geometry. Thus CFD could provide useful insights into the hemodynamics of inserted PIVCs and help identify combinations of geometry and flow related factors that may lead to device failure. Endothelial injury and hemodynamics are two aspects of Virchow’s Triad. By studying the effects of the inserted PIVC on the surrounding venous flow and the damage caused by the infusion of a secondary fluid, it could be possible to investigate ways to minimize local trauma in the vein, in particular, to reduce the WSS on the endothelial surface. *In vivo* studies have revealed the critical shear stress above which significant endothelial damage occurs^15^ and we also know that local regions of low flow and stasis, and regions of high flow and turbulence, are both important for thrombosis and vessel damage^16^. Therefore, an understanding of *in vivo* PIVC shear stresses will help better understand the causes of vessel damage.

The purpose of this study is to contribute to the science of vascular access and assist vascular access clinicians to reduce PIVC failure rates. Our aim is to establish initial data regarding which parameters are of greatest relevance to PIVC failure and provide biomechanical insights as to why device failure may occur. We achieved this by implementing a three dimensional (3D) CFD model whereby we simulated the infusion of saline into a cephalic vein under a range of clinically-relevant PIVC scenarios. The resulting data has enabled us to elucidate the hemodynamics of this widely used invasive device and help better understand some of the mechanistic reasons for PIVC failure.

## Results

### Hemodynamics of PIVCs

#### Mass fraction of blood

**Figure 1:**
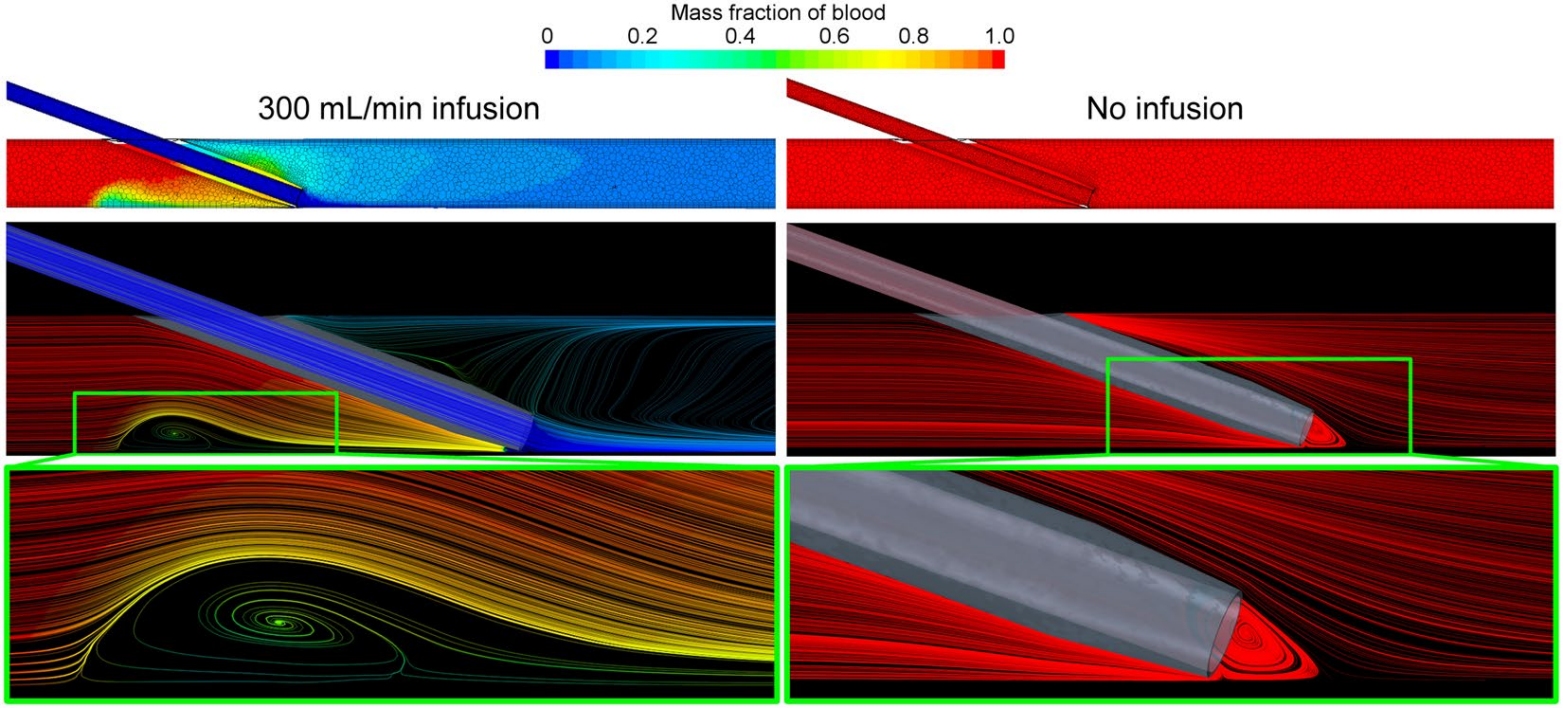
Resulting mass fraction of blood demonstrating the infusion of saline into the vein (left column) and the scenario without infusion (right column). Geometry shown is the 20 gauge (G), 20° insertion angle, positioned at the vessel edge. Only part of the geometry is shown and the images show a plane central through the catheter-vein geometry. The color bar refers to both the contours in the top row and the streamlines shown throughout. At this infusion rate (300 mL/min) the vein is practically cleared of blood and an area of recirculation (highlighted) is observed proximal to the catheter tip. In this zone the ratio of blood to saline is approximately 50:50. In the presence of the catheter, but without saline infusion, a region of recirculating blood (highlighted) is seen at the tip of the catheter.

The resulting mass fraction of blood for a representative geometry, both with and without the infusion of saline, is shown in Fig. 1. At the excessive infusion rate of 300 mL/min (5 mL/s), we see that the vein is practically cleared of blood, with a mixing region of approximately 50:50 blood to saline immediately proximal to the catheter tip. The centre of this recirculation zone is 6.5 mm from the tip of the catheter. In contrast, without any saline infusion, the vein and catheter are, of course, completely filled with blood.

#### Velocity and wall shear stress

**Figure 2:**
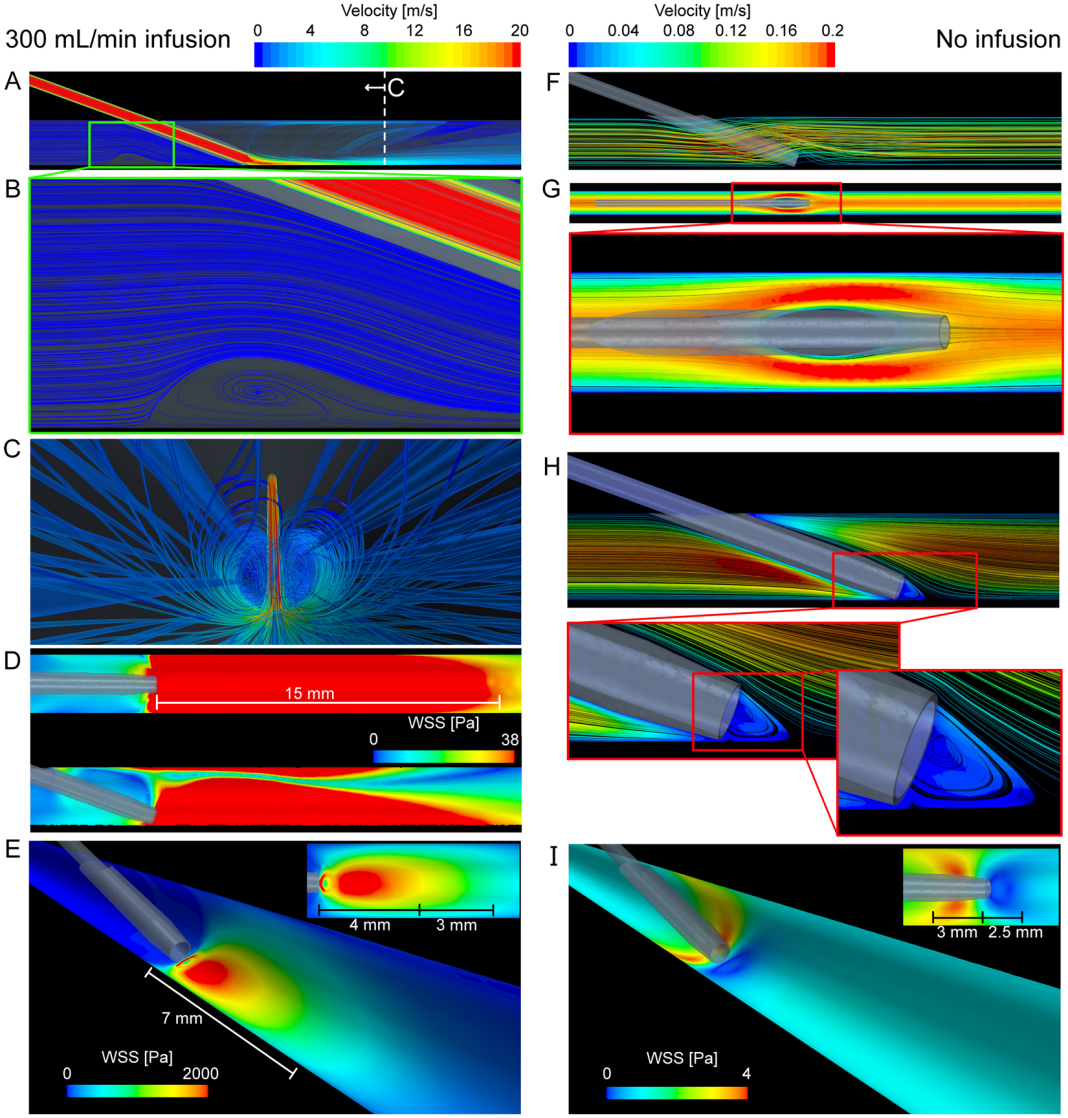
Resulting velocity streamlines and wall shear stress (WSS) due to the infusion of saline into the vein (left column) and the scenario without infusion (right column). Velocity streamlines at 300 mL/min saline infusion (**A**) show the low velocity recirculation zone (**B**) behind the catheter tip, and the internal vein view (**C**) shows the streamlines of infused saline. The view show in (**C**) is indicated in (**A**). The region of WSS > 38 Pa (**D**, top and side views shown) is an area of likely significant endothelial damage and in this scenario the WSS reaches a maximum of approximately 3800 Pa. The region of WSS > 38 Pa extends 15 mm from the catheter tip. (**E**) Contours of WSS scaled to approximately half the maximum WSS. The distance of these high WSS regions is also shown. The insert shows the view from above. Velocity streamlines (**F**) and contours (**G**, midplane of vein) of blood without saline infusion show the hemodynamics around the catheter that creates a distinct localized low velocity recirculation zone at the catheter tip (**H**). The WSS shows that without the infusion of saline, the WSS is highest behind the catheter tip and low at the catheter entrance (**I**). The distance from the catheter tip is also shown in (**I**) and the insert shows the view from above.

We use the scenario of excessive flushing through a 20 G catheter compared to when the catheter is *in situ* but without infusion for comparison (see Fig. 2). Here we can clearly see the recirculation zones described in Fig. 1, now using streamlines color-coded with velocity magnitude. In the flushing scenario, the velocity exceeds 20 m/s in the catheter and on entrance into the vein, with regions of very low velocity recirculating blood and saline behind the catheter. WSS contours show significant forces applied to the endothelium extending from the catheter tip. *In vivo* studies show that the critical shear stress of endothelial cells is approximately 38 Pa (380 dynes/cm^2^)^15^. At this level of shear stress, for even short time frames (<1 hour), endothelial cells undergo significant injury. The extreme WSS levels in the present study are dependent on infusion rate, catheter gauge and tip position and in the geometry shown in Fig. 2D, the region of potential injury (WSS > 38 Pa) encompasses the 15 mm of the vessel proceeding from the catheter tip. In contrast, when the catheter is present without saline infusion, we see that the venous blood flows around the catheter creating a region of low velocity recirculating blood directly at the catheter entrance.

### Effects on Wall Shear Stress

#### Due to catheter infusion rate

**Figure 3:**
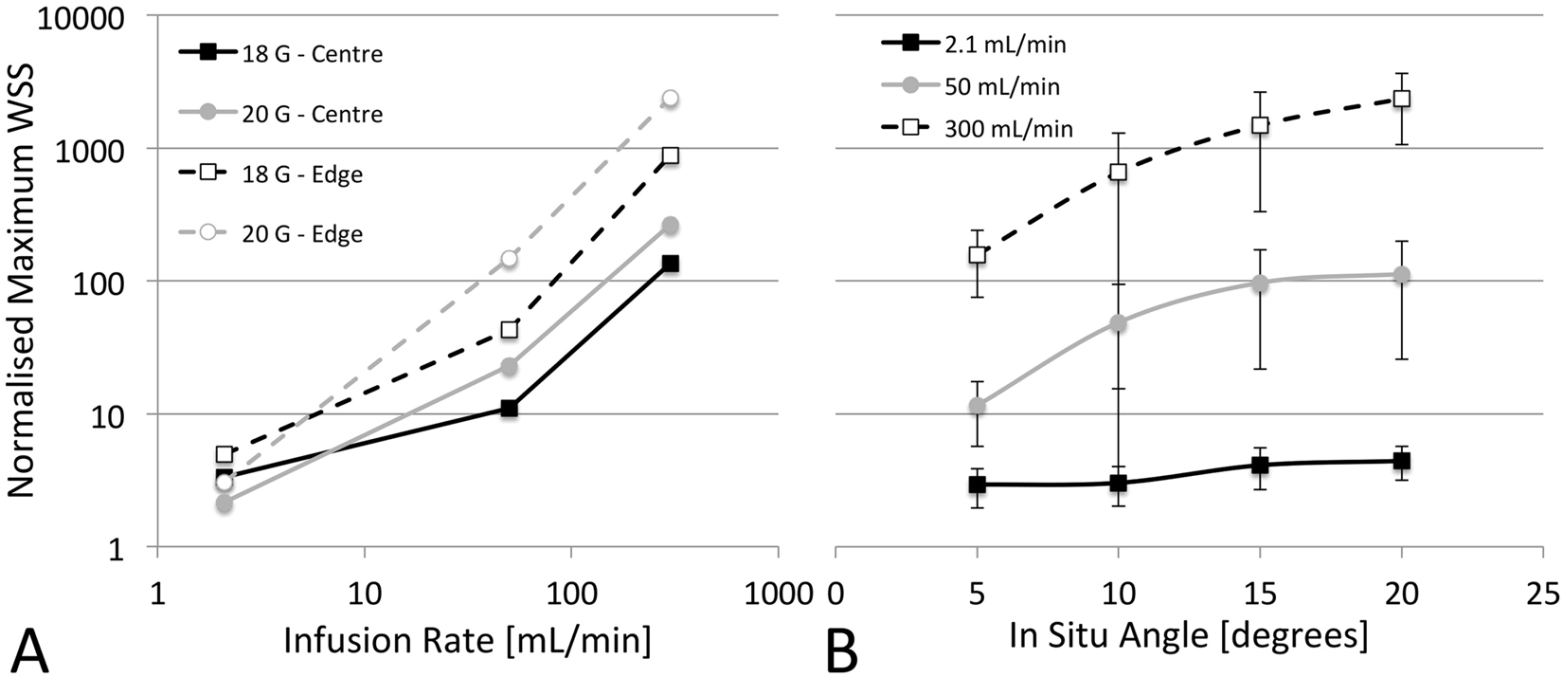
Effects of catheter infusion rate (**A**) and *in situ* angle (**B**) on wall shear stress (WSS). WSS is normalized to that of the patent vein (1.2 Pa) and presented on a log scale. Infusion rate also shown on log scale in (**A**).

The catheter infusion rate had the largest single effect on WSS (η^2^_G-Edge_ = 0.979, η^2^_G-Centre_ = 0.992, both p < 0.001, c.f. highest other η^2^_G_ = 0.898). Significant interaction effects existed between infusion rate and angle, position and gauge, as well as with position and gauge (3-way interaction). As can be seen in Fig. 3A, there is a rapid rise in normalized WSS between the medium and high infusion rates for both centre and edge positions. However, this was less evident in the 20 G catheter positioned at the vessel edge where the increase was almost linear. Between the low and medium flow rates, WSS rises faster in the edge position than in the central position. The highest WSS was seen in the 20 G, 20**°** angle, edge position configuration which had WSS 3775-times higher than that of the patent vein (3254.3 vs. 1.2 Pa)

#### Due to catheter tip position

As expected, tip position had a significant effect on normalized WSS (Fig. 3A), second only to infusion rate (η^2^_G_ = 0.949, p < 0.001), with values of WSS on average 688% higher in the edge position than central (669.2 vs. 84.9 Pa). This was highly dependent upon the infusion rate as at the lowest flow rate the increase was only 46% (4.7 vs. 3.2 Pa) compared to 716% increase (1893.0 vs. 231.8 Pa) at the highest infusion rate.

#### Due to catheter angle

Catheter angle had a significant effect on WSS for both edge and centre tip positions (η^2^_G-Edge_ = 0.728, p < 0.001; η^2^_G-Centre_ = 0.804, p < 0.05). It also had a significant interaction with infusion rate, where the effect strength increased with increasing infusion (η^2^_G-2.1_ = 0.693, p < 0.05 vs. η^2^_G-300_ = 0.847, p < 0.01). The changes in normalized WSS due to catheter angle are shown in Fig. 3B. The increase in WSS due to an increase in angle from 5**°** to 20**°** for the slow, medium and fast infusion rates was 1.5-times (4.4 vs. 2.9 Pa), ~10-times (112.6 vs. 11.5 Pa) and ~15-times (2345.7 vs. 157.3 Pa), respectively.

#### Due to catheter size

The catheter size also had an effect on WSS (η^2^_G-Edge_ = 0.855, p < 0.001; η^2^_G-Centre_ = 0.897, p < 0.01), but significant interactions exist with infusion rate where it has an opposite effect at different infusion rates. The changes in normalized WSS due to catheter gauge are shown in Fig. 3A. At the slowest infusion rate, a larger catheter increased the WSS by a combined 59% (4.8 vs. 3.0 Pa), whereas at the fastest infusion rate, the larger catheter reduced WSS by ~1.6-fold (1532.8 vs. 592.0 Pa).

### Effect on blood damage

#### Due to catheter infusion rate

The catheter infusion rate also had the largest single effect on blood damage (η^2^_G-Edge_ = 0.947, η^2^_G-Centre_ = 0.989, both p < 0.001). Interactions were present with all other parameters, but the effect of infusion rate was significant in all groups. Within both positions, infusion rate had a lower effect in the larger 18 G catheter (η^2^_G-Edge_ = 0.794, η^2^_G-Centre_ = 0.974, both p < 0.05) than in the smaller 20 G catheter (η^2^_G-Edge_ = 0.970, η^2^_G-Centre_ = 0.993, both p < 0.01).

#### Due to catheter tip position

**Figure 4:**
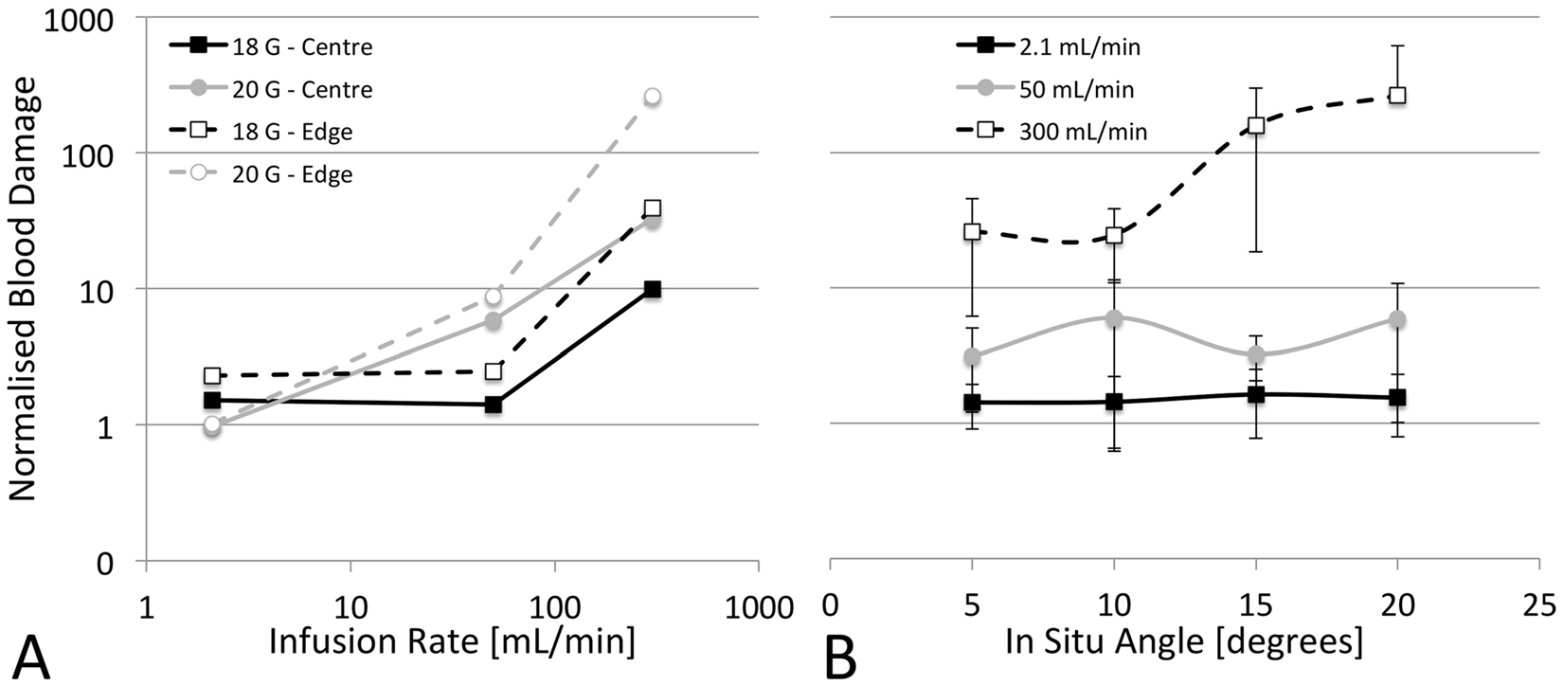
Effects of catheter infusion rate (**A**) and *in situ* angle (**B**) on blood damage. Blood damage is normalized to that of the patent vein and is presented on a log scale. Infusion rate also shown on log scale in (**A**).

The effect of position was significant for the 18 G catheter (η^2^_G_ = 0.904, p < 0.05) but not for the 20 G (η^2^_G_ = 0.03, p = 0.82). Similarly, there was a flow interaction whereby at low infusion rates, the tip being positioned near the edge only approximately doubled the damage, while at the high flow rate it increased ~5.6-times. As shown in Fig. 4, there is a relatively small effect of tip position, especially at slower infusion rates, however the angle makes a large difference. With the greater factor being flow *speed* (function of flow rate and catheter size), the effect of tip position is minimal.

#### Due to catheter angle

Catheter angle also had a significant effect on blood damage in both tip positions (η^2^_G-Edge_ = 0.786, p < 0.001; η^2^_G-Centre_ = 0.681, p < 0.05). There were, however, significant interactions with both flow and gauge. In the centre tip positions, the angle effect becomes insignificant (perhaps due to the small sample size) but shows the 20 G catheter and the 300 mL/min infusion rates has greater effect, thus dictating the trend. In the edge tip positions, catheter angle is significant for the 20 G size (η^2^_G_ = 0.905, p < 0.001) but not the 18 G size (η^2^_G_ = 0.138, p = 0.42). This effect was once again interacting with flow, specifically, the fast infusion rate. The only significant effect of angle is on the fastest infusion rate in the higher angle edge positions (10–20°) (Fig. 4B). It is likely this interaction between gauge and flow is due to the primary driver being flow speed. Therefore, at very high infusion rates, an increase in catheter angle causes an increase in blood lysis rate, but at slow flow rates it has no significant effect.

#### Due to catheter size

Catheter size has a similar effect on blood damage as it does to WSS, with significant effects at both tip positions (η^2^_G-Edge_ = 0.947, p < 0.001; η^2^_G-Centre_ = 0.989, p < 0.001), as well as significant interactions. At the lowest flow rate the larger 18 G catheter doubled the level of damage, whereas at the highest flow rate the 18 G catheter reduced the level of damage five-fold.

### Effect on Stasis Volumes and Residence Times

#### Due to catheter infusion rate

**Figure 5:**
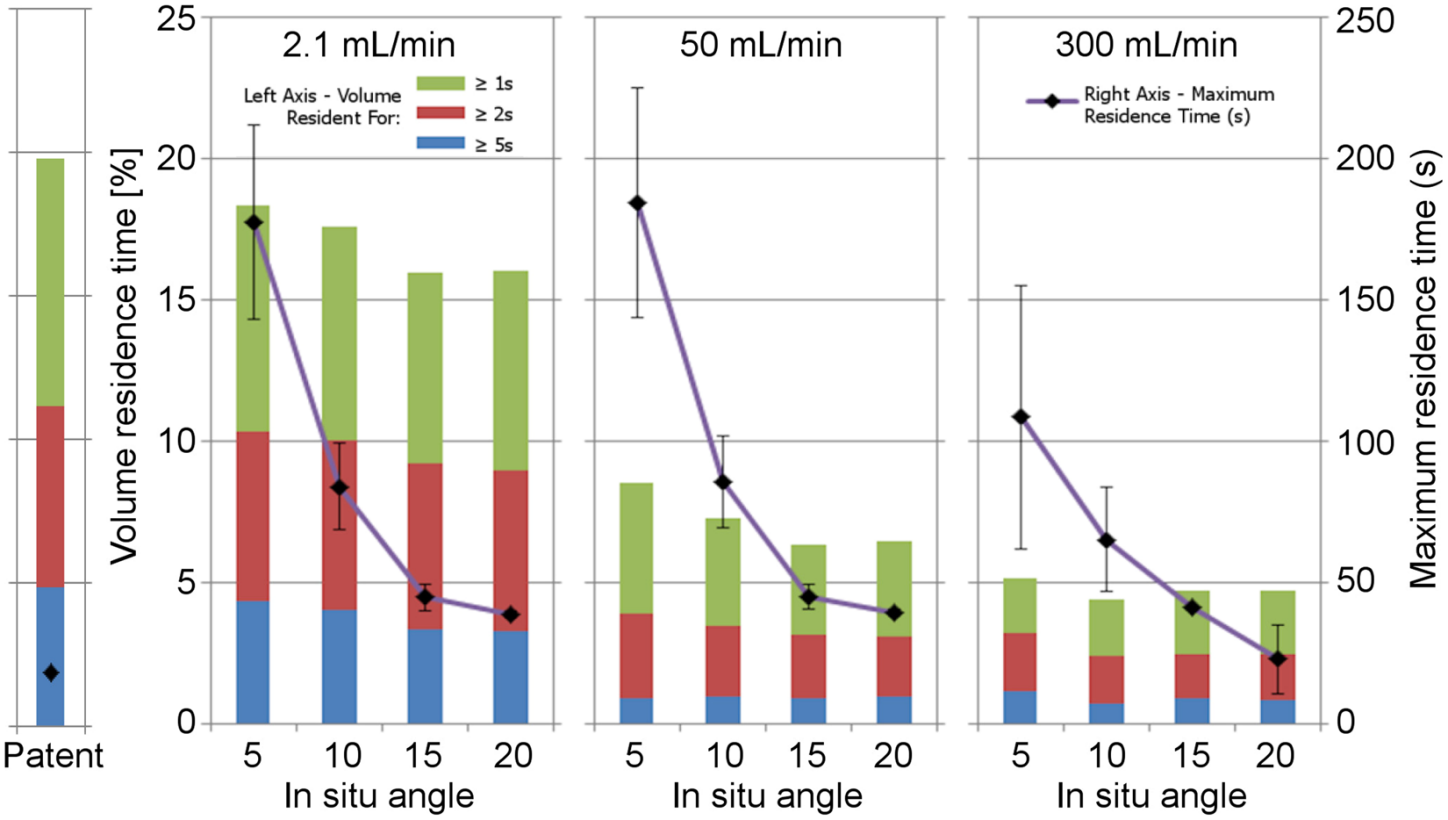
Effect of infusion rate and *in situ* angle on venous stasis volume and residence time for both catheter sizes combined.

The catheter infusion rate was the only variable of significance when measuring venous stasis volumes (η^2^_G-Edge_ = 0.41~0.53, p < 0.01~0.05; η^2^_G-Centre_ = 0.41~0.64, p = NS, c.f. highest other η^2^_G_ = 0.01). As there were no interactions present with other variables, and the effect sizes were consistent, we then averaged the flow rates and present them in Table 1 and Fig. 5. With the exception of the >5 s stasis volumes, it can be seen how these volumes significantly reduce as the infusion rate increases.

**Table 1:** Effect of infusion rate on venous stasis volume for each of the three time thresholds studied.

Infusion rate [mL/min]	Stasis volume [%]		
	>*1 s*	>*2 s*	>*5 s*
Patent vein	19.8	11.1	4.8
2.1	17.1 ± 0.4	9.7 ± 0.2	3.8 ± 0.2
50	7.1 ± 0.3	3.4 ± 0.1	0.9 ± 0.2
300	4.6 ± 0.2	2.6 ± 0.2	0.8 ± 0.1

Unlike other measures, the catheter infusion rate had relatively less effect on the maximum residence time, with major differences in effect size observed between the two tip positions (η^2^_G-Edge_ = 0.273, p = 0.08; η^2^_G-Centre_ = 0.936, p < 0.01). While the flow effect size in the centre position was high, it is almost exclusively due to a large decrease between the medium and highest flow rates. As the infusion rate increases from 2.1 mL/min to 50 mL/min, the maximum residence time remains largely unchanged as the same particles are trapped in the lee of the cannula near the insertion site. As the infusion rate is further increased, however, significant turbulence is created as the Reynolds number passes through the transition region (Re > 2600) and a recirculation zone forms in the stream, helping to clear the trapped particles. While this recirculation zone increases the residence time for entrapped particles, it does not appear to have a lasting influence due to its highly turbulent nature, and particles inevitably exit and continue downstream. This effect of flow is not evident in the edge conditions due to the angles being greater, causing a less significant acute lee region behind the cannula insertion point.

#### Due to catheter tip position

Tip position had no effect on venous stasis volumes (η^2^_G_ = 0.00~0.01, p = 0.83~0.89) or maximum residence time (η^2^_G_ = 0.02, p = 0.77). There were however, interactions with both infusion rate and catheter gauge with residence time. In the 20 G simulations, tip position continued to show no effect, but the damage was a combined ~2.5-times lower in the edge position. However in the 18 G catheter, effect size increased (η^2^_G_ = 0.94, p < 0.05) with a combined one-fold reduction in maximum residence time in the edge positions.

#### Due to catheter angle

Catheter angle had no effect on stasis volumes at either tip positions (η^2^_G-Edge_ = 0.00, p = 0.87~0.92; η^2^_G-Centre_ = 0.00~0.01, p = 0.88~0.94), but had the largest effect of any parameter on maximum resident time (η^2^_G-Edge_ = 0.881, p < 0.001; η^2^_G-Centre_ = 0.975, p < 0.001). As shown in Fig. 5, increasing insertion angle slightly decreased residence volume time but exponentially decreased maximum residence time.

#### Due to catheter size

Catheter size had no effect on stasis volumes at either tip positions (η^2^_G-Edge_ = 0.00~0.01, p = 0.75~0.92; η^2^_G-Centre_ = 0.00, p = 0.87~0.95), but did have an effect on maximum residence time (η^2^_G-Edge_ = 0.476, p < 0.05; η^2^_G-Centre_ = 0.945, p < 0.01). The larger 18 G catheters (outer diameter = 1.27 mm) reduced maximum residence time by a combined average of 45%.

### Infusion Rates and Wall Shear Stress

**Figure 6:**
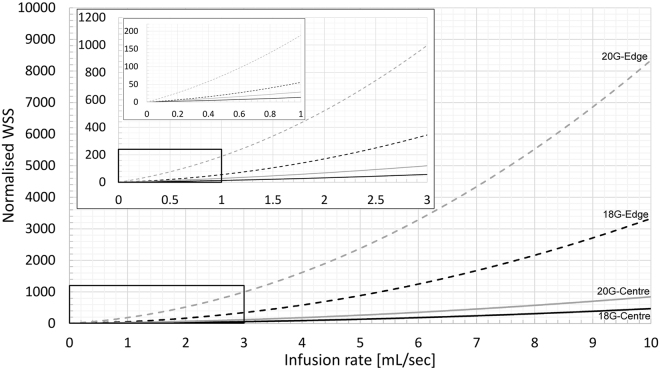
Effect of infusion rate through a 18 and 20 G catheter positioned centrally or at the vessel edge on the normalized wall shear stress in the vein.

In order to provide some practical indication of what the combined effects of infusion rate, catheter size and tip position imply, in Fig. 6 we show the normalized WSS for a range of infusion rates. The models used to generate these WSS data for each catheter and tip position are shown in Equations 1–4, where *x* is the infusion rate in mL/s. In practice, the optimal volume and frequency of flushing is unclear, with current guidelines suggesting flushing with twice the volume of the catheter plus any other devices. Our own observations are that 1 mL/s is safe in practice among adult populations and reflects approximately 3–10 mL flushed over 3–10 seconds. However, in emergency medicine and interventional radiology, rapid infusion above this rate could be envisaged. From Fig. 6 we can make some approximate observations about potential endothelial damage. For example, when a 20 G catheter is at the edge of the vessel, even at low infusion rates (above 0.3 mL/s), the WSS is 40 times the value we calculated for the normal patent vein (1.2 Pa). We can also use these models to approximate the infusion rate for each catheter that exceeds the critical endothelial shear stress of 38 Pa reported by Fry^15^. For the 18 G and 20 G catheter positioned centrally, the infusion rate that reaches this critical threshold is 2.5 mL/s and 1.4 mL/s, respectively. Whereas for the 18 G and 20 G catheter positioned at the vessel edge, the infusion rates are 0.85 mL/s and 0.3 mL/s, respectively.

1 $$18\,{\rm{G}}\mbox{--}{\rm{central}}\,{\rm{position}}:\quad \quad {WSS}=4.0746{x}^{2}+6.0725x+3.1187$$

2 $$18\,{\rm{G}}\mbox{--}{\rm{edge}}\,{\rm{position}}:\quad \quad {WSS}=31.182{x}^{2}+20.322x+4.196$$

3 $$20\,{\rm{G}}\mbox{--}{\rm{central}}\,{\rm{position}}:\quad \quad {WSS}=6.4093{x}^{2}+20.513x+1.4294$$

4 $$20\,{\rm{G}}\mbox{--}{\rm{edge}}\,{\rm{position}}:\quad \quad {WSS}=71.599{x}^{2}+117.92x-1.1518$$

## Discussion

In this computational study of PIVCs, we have shown the effects of infusion rate, catheter angle, position and size, on WSS, blood damage, residence time and venous stasis. We report the first data on the interplay between routine PIVC parameters and the resulting hemodynamic environment that may both contribute to device failure. PIVC failure not only requires reinsertion of a new catheter, but also leads to several complications for the patient. As PIVCs are one of the most widely used medical devices, and up to 50% fail, a better understanding of the controllable mechanisms of failure is of major clinical significance.

We show that the presence of the catheter and the infusion of fluid (e.g. saline) has a dramatic impact on WSS, and in some configurations increasing WSS by over 3000 times that found in the patent vein (~3800 Pa in the 20°, 300 mL/min, 20 G condition vs. ~1.2 Pa in the patent vein). This level of WSS has the potential to remove endothelial cells^15^ and may initiate biological responses. WSS was most greatly affected by catheter infusion rate however catheter tip position, gauge and insertion angle all had significant effects on WSS, in descending order, respectively. For PIVCs positioned at the vessel edge, our simulations (Figs 1 and 2) reveal a low velocity recirculation zone of mixing blood and saline behind the catheter tip. We also see that the presence of the catheter without any infusion, significantly changes the flow field and results in a localized low velocity recirculation zone with low WSS at the catheter tip (Fig. 2). It remains to be determined whether such recirculation results in platelet aggregation, fibrin deposition and thrombus formation in PIVCs. However, we do know that low WSS will cause thrombosis^17^ so it is likely that the low WSS region here plays a role in catheter occlusion if the catheter remains *in situ* for prolonged periods of time.

The presence of the catheter and saline infusion also affected the level of blood damage, with values up to 510 times that found in the patent vessel. As with WSS, catheter infusion rate had the largest impact, followed by catheter gauge and *in situ* angle. Tip position did not have a significant effect on blood damage (p = 0.051), yet could be due to the sample size used here as the effect size was still moderate (η^2^_G_ = 0.65). Our data indicates that the tip position had no significant effect for the smaller catheter, but had a moderate effect for the larger catheter. Interestingly, at an infusion rate of 2.1 mL/min through a 20 G catheter, blood damage did not increase compared to the patent vessel, in any configuration. However the same flow rate through the 18 G catheter approximately doubled the blood damage in all geometric configurations.

In the control vein without a catheter, large volumes of slow traversing blood (for each of the 1 s, 2 s and 5 s thresholds) were observed. The introduction of the catheter decreased these volumes, with infusion rate the only parameter affecting stasis volume. The slowest infusion rate reduced volumes by about 5%, while the fastest infusion rate reduced volumes by about 15% of the control vein. The maximum residence time, however, was low in the control vessel (18.7 s) compared to all of the vessels with PIVCs (34.8–225 s), peaking in the 5° angle simulations (20 G, 5° angle, centrally positioned, 50 mL/min infusion). The catheter angle had the greatest influence on residence times, with increasing angle exponentially decreasing maximum residence time in all infusion rates. The increased insertion angle increases the area in the lee region of the catheter, and so fewer particles can become trapped.

Our data suggests it is the *speed* of the fluid being infused that is likely a critical factor dictating damage, compared to the individual infusion flow rate or gauge combinations. This is to be expected as faster moving fluid needs to decelerate more when joining a vessel, transferring kinetic energy to either the vessel wall or the blood volume, both causing shear stress and damage to either the vessel wall or to the blood particles. The mechanisms for residence time, however, are different. The maximum residence time is largely caused by blood pooling in the lee of the catheter near the insertion point, therefore geometry is the critical factor, with large volumes of incoming turbulent flow only partially able to assist by circling back to dislodge these particles. Similarly, the blood volumes resident for long periods of time are reduced by the addition of high volumes of flow, which act to increase the flushing through the vessel. As such, for particle residence time, peak speed is less important than the overall quantity of flow that is pushed through the vessel.

Nifong and Devitt^12^ provided useful data on the effect of catheters located centrally in veins. They show that the rate of fluid flow is significantly correlated to catheter size. However, their model simulates a centrally-positioned object in a vessel; they did not study the angulation of the *in situ* catheter and the infusion of a secondary fluid into the flowing blood. They also did not investigate shear stress. These aspects are difficult to study without using more sophisticated computational methods such as those used here. CFD has been used to model the flow field near a venous needle in hemodialysis, where the influence of needle position and flow rate within an artery, and the resulting effects on WSS, were reported^18^. Furthermore, Ghata *et al*.^19^ simulated the effect of blood flushing with saline with a view to optimizing flushing parameters for clearing blood from a vessel. Our study uses CFD, with turbulence models where relevant, to show how routinely used PIVCs and infusion rates impact both the blood and the vein wall, and how these measures can be used to better understand why PIVCs may fail and potentially inform new clinical practice.

We show that WSS and blood damage follow broadly similar trends and tip position influenced both these measures due to the redirection of force into the wall. However, to understand which measure is more important clinically, ultrasound imaging could be used to identify different thrombus formation zones. Thrombus formation at the vessel wall immediately downstream of the PIVC tip is likely indicative of wall damage induced by supraphysiological shear stress, perhaps from an excessively fast flush or, as is often the case, an irritant drug. There have been many studies showing the correlation between irritant drugs and PIVC failure^20^, and avoiding the contact between high concentrations of irritants and the endothelial lining is essential to preventing chemical instigation of damage. In contrast, thrombus development further downstream could result from the damaged blood particles traversing the vein before a response is mounted. Additionally, thrombosis due to venous stasis would likely cause occlusion in the lee of the catheter, near the insertion site, though this is not common clinically.

From our own clinical observations we know that thrombosis frequently occurs downstream, close to the tip of the catheter, implicating damage to the wall as the primary trigger of device failure. In our models, especially those of excessive infusion rates, we see that the region of high WSS extends distally from the catheter tip, with the length of this region dependent on the infusion rate and catheter-vein geometry. In the configuration shown in Fig. 2, the high WSS zone begins at the catheter tip and encompasses the proceeding 15 mm of vein (Fig. 2D). This agrees well with clinical observations stated previously. Thus it is likely that the excessive WSS causes venous wall damage, leading to phlebitis and thrombosis, and ultimately to PIVC failure. Whereas, with catheters *in situ* for periods of time without infusion, the flow field around the tip is likely to be the primary cause of occlusion. Current Australian guidelines vary, but suggest PIVC removal if not used for 12–24 hours. More research is required to understand if PIVC removal is beneficial or detrimental to overall patient outcomes, and removal/reinsertion may not be necessary in many cases. However, if we are to try reduce PIVC failure rates, alternative strategies are needed and PIVC removal is the most certain way to reduce the chance of PIVC failure.

There are, however, some limitations to our work, such as the use of idealised geometries. Although the key mechanisms we show are valid, patient-specific geometries will influence the hemodynamics and alter the strength of some of the interactions. We modeled the catheter tip as blunt instead of using a 5° taper, to focus on the overall effects of the infusion of a secondary fluid rather than on the specific flow pattern close to the tapered tip. Models of different tips would be a useful future study. Similarly, we did not investigate the effect of eyelets in the catheter^18^. We tested the most clinically-relevant parameters (i.e. catheter size, insertion angle and infusion rate) and additional iterations would add little value. Also, we have not specifically considered the physical interaction between the catheter and the vessel wall in our simulations. Modeling the motion of the catheter-vessel wall interface will give rise to stresses at the tissue-device boundary, further contributing to physiological changes and potentially, thrombosis. We assumed blood to be a Newtonian fluid. Incorporating non-Newtonian behaviour would increase the fidelity of our models, however at the vessel diameters studied here, the use of a non-Newtonian blood model is unlikely to change the overall findings. We omitted length as a variable as most catheters are a standard length (e.g. 35 mm), however, with further work the optimum positioning (i.e. centrally) could be determined by catheter length, angle, and ultrasound. Imaging could measure the distance from insertion site through the skin to the entry point in the vein. So for example, if the catheter is 35 mm long and the distance from skin surface to vein is 25 mm, it is then clear that 10 mm of catheter can be used to purchase the vein and the amount of catheter protruding into the vein will depend on the insertion angle. The implementation of turbulence models also introduces uncertainty into the excessive infusion simulations. Turbulent fluid flow, an area of on-going research, has features on several different length scales which all interact with each other. To accurately capture these interactions computationally, a direct numerical simulation (DNS) is required which is computationally costly even at low Reynolds numbers. For the Reynolds numbers encountered in most applications, the computational resources exceed what is currently possible. Finally, experimental validation is desirable, but beyond the scope of the current study. For example, ultrasound could be used to measure the flow within the vein while also infusing into a nearby PIVC. This imaging could measure deformation of the vessel when bolus injections are given, and also validate the downstream flow rates. This is timely as ultrasound-guided PIVC post-insertion failure rates are similar if not worse than landmark placed PIVCs^21^.

In conclusion, we have performed a comprehensive computational study of PIVCs to better understand the hemodynamic environment likely to contribute to device failure. We show that the most important factor influencing the measures we investigated is the infusion rate in the catheter, with excessive infusion rates leading to extremely large increases in potential damage to the vessel wall and the blood itself. As such, our data suggests limiting the use of flushing speeds to below 1 mL/s and to use ultrasound to guide the catheter tip position to a central location, so as to minimize critical WSS effects and to reduce potential vessel damage. If faster infusions are required, the largest gauge catheter (centrally-positioned) suitable for the particular vein should be used to reduce ejection velocity and resulting damage. We also find that the presence of the catheter itself, even with slow infusion rates, leads to large increases in stasis times. However, without any infusion, the presence of the catheter causes flow obstruction and creates low velocity recirculation zones and low WSS regions that likely promote platelet and fibrin deposition and thrombosis at the catheter tip, potentially leading to occlusion.

## Methods

We used computational fluid dynamics (CFD) models of a peripheral vein to simulate hemodynamics in the presence of a PIVC and various infusion rates. These models allow us to vary specific controllable parameters in the clinical use of PIVC and test their impact on measures derived from fluid mechanics that are known to cause injury, such as wall shear stress (WSS). CFD solves the Navier-Stokes equations computationally and is used throughout engineering to simulate the flow of liquid or gas, and to understand how this flowing mass affects objects it flows past.

### Parameters investigated

**Figure 7:**
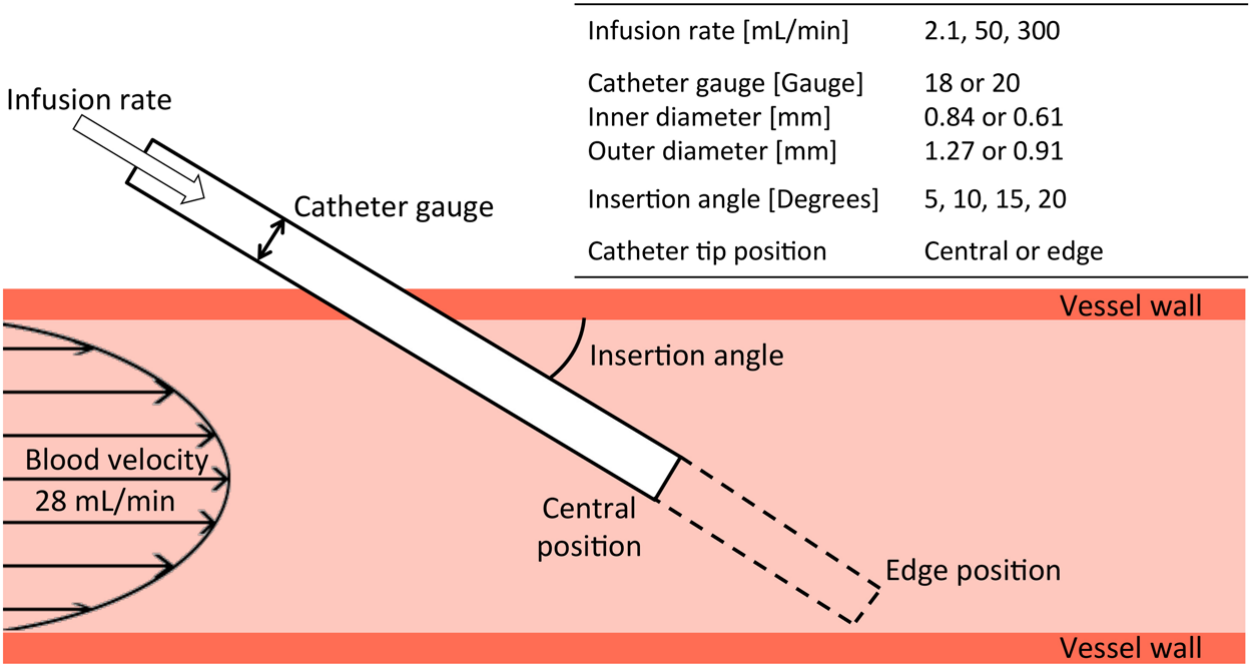
Illustration of parameters investigated.

We focused on the following parameters: impact of catheter infusion rate, catheter tip position in the vein, catheter insertion angle, and catheter gauge (diameter). These parameters are shown in Fig. 7 and described in the following sections.

#### Catheter infusion rate

Three infusion rates were selected: (a) 1 L/8 Hr (2.1 mL/min) to simulate slow rehydration; (b) 1 L/20 min (50 mL/min) to simulate rapid rehydration or infusion of drugs; (c) 10 mL/2 sec (300 mL/min) to simulate forceful ‘flushing’ to test device patency and expel any contaminants.

#### Catheter size, tip position and angle

We investigated two typical catheter sizes: 18 gauge (G, inner diameter = 0.84 mm; outer diameter = 1.27 mm) and 20 gauge (G, inner diameter = 0.61 mm; outer diameter = 0.91 mm); two tip positions (central in vein and adjacent to vein wall); and four insertion angles (5°, 10°, 15° and 20°). However, we eliminated configurations that were not clinically relevant. For instance, with a 5° insertion angle almost the entire length of the catheter would need to be inside the vein to reach the bottom edge of the vessel. Additionally, at 15° and 20° insertion angles, tip positions in the centre of the vein result in little of the catheter embedded in the vessel. Therefore we investigated five combinations of tip position and insertion angle for each catheter size and infusion rate (Table 2). In addition, we modeled the hemodynamics in the vein without the presence of a catheter to use as a control. Finally, we simulated the 20 G catheter inserted at 20° into the vein, without any saline infusion, to use as a qualitative comparison and study the effect of the catheter on the flow field.

**Table 2:** Final geometric configurations used to simulate each infusion rate.

Gauge [G]	Angle	Position
20	5	Centre
	10	Centre
		Edge
	15	Edge
	20	Edge
18	5	Centre
	10	Centre
		Edge
	15	Edge
	20	Edge

### Computational fluid dynamics simulations

We implemented a 3D, steady state, multi-component Newtonian liquid, non-reacting, coupled flow, coupled species, constant density, computational approach. We used a laminar model for the lower infusion rates and a Reynolds-Averaged Navier-Stokes (RANS) turbulence model for the 300 mL/min infusion rate. All computational work was performed using STAR-CCM+ (v11.04, CD-adapco, Siemens).

#### Geometry creation

We assumed the vessel was a 100 mm long, straight cephalic vein with a diameter of 2.4 mm and rigid walls. We omitted any venous valves in our vein geometry. The catheter was inserted to either the central or edge position at 5**°**, 10**°**, 15**°** or 20**°** with respect to the vein. All geometries were created using the computer-aided design tools in STAR-CCM+.

#### Computational model

**Figure 8:**
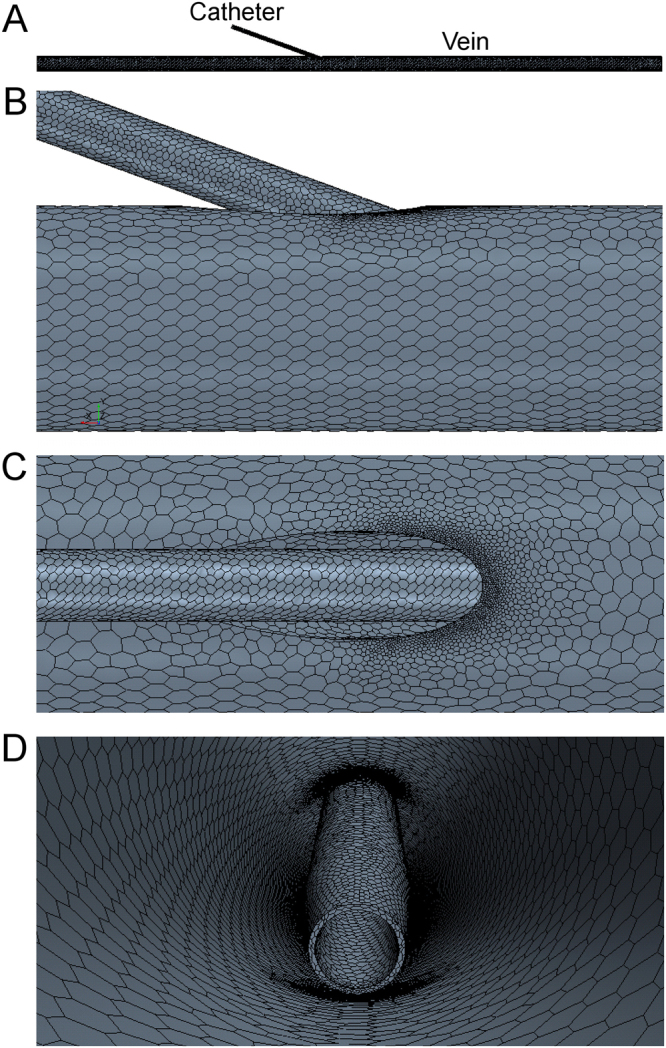
Representative computational mesh on the 20 G, 20° insertion angle, positioned at the vessel edge. (**A**) Entire domain showing catheter inserted into vein. Close-up of mesh showing refinement around insertion region from the side (**B**), top (**C**) and internal (**D**) views. Element size was reduced inside the catheter and in catheter-vein regions.

We created a computational mesh of polyhedral elements with a maxumum edge size of 100 μm within the catheter and in the catheter-vein region, increasing to 200 μm throughout the vein. Eight layers of prism elements with a total thickness of 100 μm, were biased towards the wall. This mesh refinement was used to capture the steep velocity gradient in the near-wall boundary layer. The computational mesh is shown in Fig. 8. Both blood and saline were assumed to be Newtonian fluids with densities of 1050 kg/m^3^ and 1005 kg/m^3^ and viscosities of 2.78 Pa.s and 1.02 Pa.s, respectively. The vein inlet velocity was assumed to be 28 mL/min and was applied as a parabolic function. This flow rate was measured using Doppler ultrasound in a previous study^22^. Infusion rates through the catheter are stated earlier and represent those used clinically. The vein inlet was set to 100% blood and the catheter to 100% saline. In addition to the RANS turbulence model for the 300 mL/min infusion rate simulations, we used the Mentor’s Shear Stress Transport (SST) K-Omega turbulence model as it incorporates the K-Epsilon model’s ability to handle free-stream conditions, with the K-Omega model’s ability to handle near-wall conditions. Turbulence parameters (length and intensity) were calculated from the known flow speeds in the catheter and vein as a result of the infusion of 300 mL/min of saline (Re > 10,000 inside the 20 G catheter; Re ~2500 in the vein). Reynolds number (Re) indicates the presence of turbulent flow phenomena. The vein outlet was set to zero pressure and we assumed the non-slip condition at the vessel and catheter rigid walls.

### Hemodynamic metrics

We began by calculating WSS from our simulations. Excessive WSS damages the endothelial lining of the vessel^15^ causing inflammation (phlebitis), whereas low WSS that occur in regions of low velocity can lead to thrombus formation^17^. We then studied blood damage (throughout this work we use the term blood damage to represent hemolysis), a surrogate measure of red blood cell lysis, which may cause activation of platelets, leading to thrombus formation. We measured the residence time of blood particles and volumes of venous stasis where we chose 1, 2 and 5 second thresholds for stasis volumes. Residence time and stasis volumes allow us to investigate the proportion of fluid in the system that takes a long time to exit. In order to implement the residence time and blood damage calculations, we used two passive scalars, one for residence time and one for the damage scalar. We followed the work of Garon and Farinas^23^ to implement the damage scalar *σ*, which is defined in Equation 5, where *τ* is the WSS.

5 $$\sigma ={(3.62\times {10}^{-7})}^{\frac{1}{0.785}}\cdot {\tau }^{\frac{2.416}{0.785}}$$

Equation 5 returns a result applicable over either 2D or 3D domains that was shown to produce high correlation with measured lysis rates in calf blood flowing through an experimental apparatus^23^. We modified Equation 5 to account for the reduced quantity of red blood cells in downstream region as the saline diffuses. To achieve this we multiplied *τ* by the mass fraction of blood so the strain rate affects the particles in the saline at a proportionate rate, reducing that experienced by the red blood cells.

### Data analysis

WSS and blood damage values were normalized using simulation data from the vein without any catheter. As the study was performed with only a fractional-factorial design, the results were analysed using ANOVA on subsets of the data (using between-subject effects). For the effects of catheter flow rate, angle and catheter gauge, two ANOVAs were run independently on both the centre tip position (5° and 10° angles) and edge tip position (10°, 15° and 20° angles) cases. For the effects of position, only the 10° angle was used as it is the only angle to include both tip positions. We measured the effect size using the generalized eta-squared (η^2^_G_)^24^ and determined p-values using F-tests. The subscripts accompanying η^2^_G_ indicate either the tip position or the infusion rate. We deemed statistical significance when p < 0.05. To provide practical information on the effects of infusion rate, catheter size and position, we fit polynomial equations to the normalized WSS data and extrapolated the data to higher infusion rates (to a maximum of 600 mL/min (10 mL/s)).

### Data availability

The datasets generated as part of this study are available from the corresponding author on reasonable request.
